# Understanding the Neuropsychological Implications of Klinefelter Syndrome in Pediatric Populations: Current Perspectives

**DOI:** 10.3390/pediatric16020036

**Published:** 2024-05-25

**Authors:** Panagiota Tragantzopoulou, Vaitsa Giannouli

**Affiliations:** 1 School of Social Sciences, University of Westminster, London W1W 6UW, UK; 2 School of Medicine, Aristotle University of Thessaloniki, 54124 Thessaloniki, Greece; giannouliv@hotmail.com

**Keywords:** Klinefelter syndrome, neuropsychology, memory, language, mental disorders

## Abstract

Klinefelter syndrome (KS), also known as 47,XXY, is a genetic disorder characterized by the presence of an extra X chromosome. Despite the prevalence of verbal learning disabilities, memory impairments, and executive function deficits in individuals with KS, comprehensive research on the neuropsychological profiles of affected children and adolescents remains limited. Additionally, KS has been associated with comorbid conditions such as depression, anxiety, schizophrenia, attention-deficit hyperactivity disorder (ADHD), and autism spectrum disorders (ASDs). However, systematic investigations into the neuropsychological manifestations of KS in pediatric populations are scarce. Therefore, the primary objectives of this review are to provide an overview of key studies examining the neuropsychological profiles of children and adolescents with KS and to delineate the limitations and implications of existing research findings. By synthesizing available literature, this review aims to bridge the gap in understanding the cognitive and behavioral characteristics of children and adolescents with KS, shedding light on potential avenues for future research and clinical interventions. Ultimately, this review serves as a valuable resource for clinicians, researchers, policymakers, parents, and educators involved in the assessment and management of the neuropsychological aspects of Klinefelter syndrome in pediatric populations.

## 1. Introduction

Klinefelter syndrome (KS) is a prevalent condition that leads to health issues such as testicular failure, androgen deficiency, and impaired spermatogenesis. While KS is relatively common, occurring in approximately 1 in 600 males with the 47,XXY karyotype, it is seldom identified during childhood and adolescence, and a significant proportion remain undiagnosed throughout their lives [1]. This condition was initially identified in 1942 by Klinefelter et al. [2] who observed a group of nine men displaying symptoms including gynecomastia, small testes, azoospermia, and elevated gonadotropin levels. Their research suggested that the primary cause of hypogonadism in these individuals was a malfunction of the Sertoli cells in the testes, responsible for spermatogenesis. Interestingly, they noted relatively normal functioning of Leydig cells, responsible for testosterone production, as indicated by the appropriate distribution of pubic and axillary hair. Additionally, Klinefelter and colleagues hypothesized the presence of a secondary testicular hormone, potentially regulating pituitary gonadotropin levels via feedback inhibition, which they tentatively labeled as X hormone or inhibin [2].

In 1949, Barr and Bertram made a significant discovery regarding the presence of dense chromatin masses, later termed Barr bodies, exclusively in the nerve cell nuclei of female cats [3]. This finding sparked interest in exploring the presence of Barr bodies in human somatic cells, particularly in females. As a result, researchers began using stained smears of buccal mucosal cells to discern an infant’s genetic sex by examining the presence or absence of Barr bodies, which typically denote the female sex [4,5]. By 1956, two separate groups of investigators utilized buccal smear analyses to identify seven patients with KS, characterized by the presence of Barr bodies [6,7]. Subsequent research in 1959 confirmed the correlation between KS and an additional X chromosome, establishing a karyotype of 47,XXY [8]. Further studies unveiled various X chromosome abnormalities associated with KS, including 48,XXXY and mosaicism, where different cell lines in an individual exhibit varying chromosomal compositions [9,10]. Notably, in cases of mosaicism, Barr bodies may not be detectable in buccal smear results due to the possibility that the extra X chromosomes are localized in tissues other than buccal cells, such as testes or peripheral blood cells [10].

After this brief historical introduction to the syndrome, the subsequent sections of this review will examine and analyze the existing literature concerning the symptoms and the neuropsychological implications of KS in pediatric populations (see Table 1). The discussions will center on various aspects, including executive dysfunction, language deficits, clinical challenges such as psychiatric disorders and social impairments, as well as other associated challenges. Furthermore, guidance for future interventions and research directions will be provided, addressing the needs of researchers, clinicians, parents, educators, and policymakers in this field.

## 2. Neuropsychological Profile

### 2.1. Executive Dysfunction

One area of particular concern in the pediatric population diagnosed with KS revolves around executive function, which includes processes such as information processing speed, selective and sustained attention, response inhibition, working memory, cognitive flexibility, conceptualization, initiating, planning and goal setting [11]. Limited research on executive function in children with KS suggests consistent deficits in tasks with significant verbal and/or motor components. Previous studies found mixed results, with some showing an intact performance on certain tasks like the Wisconsin card sorting test, but an impaired performance on others like the trail making test [12,13,14,15,16]. Additional research indicates that boys with KS perform worse on tasks such as verbal fluency, trail making, and spatial working memory compared to boys without XXY [17]. However, a reliance on recruitment from parent support groups in this study may introduce bias due to prenatal and postnatal diagnoses, potentially affecting generalizability. Additionally, the relatively privileged socioeconomic backgrounds of both the KS and control groups may limit the applicability of the results to the broader population. Nonetheless, Tran et al. [18], in a sample of 111 males, confirmed that boys with KS often present low working memory capabilities, which can be mediated with hormone replacement therapy (HRT) if started within the first 5 years of life. Despite these valuable insights, the study encountered limitations due to inconsistent bloodwork results, impacting the availability of androgen level measurements both before and after HRT.

An additional study aimed to address the Issue of finding generalizability by examining executive functions in a diverse sample of boys and adolescents with KS (mean age = 12.5 years old). Using a comprehensive battery of performance-based measures, including The Wechsler Scales of Intelligence and The Delis Kaplan Executive Function System, as well as parent-rating scales, the study assessed executive functions in 77 participants [19]. The results showed that many of the boys and adolescents had difficulties with attention and thinking skills, even though their intelligence scores were found to be in the average range. Parents also reported challenges related to initiation, working memory, planning, organization, and monitoring in their children. Additionally, the study investigated executive functions in participants with and without an attention-deficit/hyperactivity disorder (ADHD) in the XXY sample. Surprisingly, boys with KS and ADHD did not perform worse on the tests compared to those without ADHD [19], which contrasts with the findings from studies of developmental ADHD [20]. This suggests that executive function deficits may be inherent to the XXY phenotype, rather than solely attributable to an ADHD diagnosis. Green et al. [21] also found that boys with KS showed attention problems and working memory weaknesses compared to typically developing children, presentations that were thought to resemble ADHD symptoms.

Despite recognizing the detrimental impact of executive dysfunction on long-term academic, adaptive, and psychological functioning [22,23], the underlying neurobiological mechanisms responsible for executive function deficits in KS remain unclear. A recent investigation aimed to shed light on executive function, brain activation patterns, and pubertal development in adolescents with KS. The study involved 43 adolescents diagnosed with KS (average age: 12.3 ± 2.3 years old) and 41 typically developing boys (average age: 11.9 ± 1.8 years old) [24]. Participants underwent a pubertal assessment, behavioral evaluation, and functional magnetic resonance imaging (fMRI) while engaging in an executive function task, specifically the go/no-go task. The results revealed that boys with KS exhibited a diminished executive function along with reduced activation in brain regions associated with executive function, including the inferior frontal gyrus, anterior insula, dorsal anterior cingulate cortex, and caudate nucleus. Further analysis indicated a correlation between the extent of activation differences in boys with KS and the severity of pubertal developmental delay, as indicated by lower levels of testosterone and reduced testes volume [24]. These findings suggest a neural foundation for executive dysfunction in KS and propose that disruptions in pubertal development may contribute to the heightened severity of this cognitive impairment. They also emphasize the complex interplay between neurodevelopmental factors, such as pubertal development and brain function, in contributing to the cognitive profile observed in pediatric populations with KS. Further research is warranted to elucidate the specific mechanisms underlying these associations and their implications for intervention and support strategies.

### 2.2. Language Impairments

Research on speech production among children and adolescents with KS remains limited, despite documented language deficits. Language difficulties encompass delays in the emergence of initial words and the attainment of key language developmental milestones, as well as challenges in various specific areas. Infants diagnosed with KS often exhibit delayed babbling and acquisition of sounds, alongside difficulties in breastfeeding, suck–swallow coordination, and the organization of oromotor musculature [25,26]. These language deficits may manifest as issues with articulating sounds or syllables, as well as difficulties in retrieving and processing phonemes during speech.

Early studies have reported articulation issues in children with KS. Yet, the lack of methodological specificity in these studies makes interpretation challenging. For instance, studies by Leonard et al. [27] and Robinson et al. [28] relied solely on school records as a measure, with their samples encompassing various aneuploidies beyond 47,XXY, complicating generalizations specific to KS. Delayed language milestones have been observed in early childhood, with a study on 18-month-old KS males revealing decreased early lexical and syntactic abilities compared to controls [29]. Further, deficits in vocabulary, grammar, and/or auditory memory have been documented, with studies showing decreased vocabulary and comprehension in KS boys compared to controls [30] and impairments in complex language processing, expressive vocabulary, verbal memory, and expressive grammar [12,31]. In contrast, some studies have reported intact receptive and expressive vocabulary but deficits in higher-level metalinguistics and pragmatics [13]. These discrepancies in the findings may stem from variations in study methodologies, sample characteristics, and the specific linguistic domains assessed, highlighting the complexity of language deficits in individuals with KS.

An additional exploration of two cases of children with KS revealed that both children exhibited weaknesses in understanding metaphors and implied meanings. Additionally, both children demonstrated difficulties in understanding social contexts. These findings suggest that children with KS may face challenges in comprehending abstract language concepts [32]. However, each child exhibited unique strengths and weaknesses, underscoring the complexity of language abilities in individuals with KS. Limitations of the study include the small sample size and the absence of control groups, which may affect the generalizability of the findings.

Reading and writing difficulties often co-occur with speech and language disorders in early life, posing significant challenges for boys with KS [33]. Although some research has identified strengths in single-word reading and phonetic decoding, significant difficulties across various reading and spelling tasks have also been observed [31,34]. A recent study involving 26 children and adolescents highlighted common communication impairments characterized by oromotor, speech, language, and pragmatic impairments, with a possible trend for more significant deficits with age and increasing academic and social demands [35]. Notably, ongoing phonological errors in speech production were identified as a new finding.

Throughout development, individuals with KS consistently demonstrate language weaknesses relative to overall cognitive performance [22], with some scholars proposing them to be similar to those observed in cytogenetically normal dyslexic children and those in the autistic spectrum disorder (ASD) [36,37]. These challenges endure into adolescence, leading to significant discrepancies in academic achievement between individuals with KS and their peers, impacting various domains, including mathematics, problem-solving, the establishment of cause–effect relationships and analogies, and overall knowledge integration [38]. As a result, a substantial proportion, ranging from 60% to 86%, of students diagnosed with KS are enrolled in special education programs [39].

These literacy and language challenges may be linked to deficits in auditory short-term memory [31,34]. Similarly, Bishop and Scerif [37] proposed as a possible explanation of the language impairments the neuroligin–neurexin (NN) hypothesis. This theoretical framework posits that language deficits observed in individuals diagnosed with KS and other sex chromosome trisomies (SCTs) may stem from disruptions within a neural network crucial for synaptic signaling, wherein neuroligins and neurexins play pivotal roles. This proposition is founded upon the known functions of these genes in modulating synaptic plasticity, as well as their association with neurodevelopmental conditions characterized by language impairment and challenges in social interaction. Further research is needed to explore the interplay between speech, language, literacy, language memory, and other cognitive processes in individuals with KS, shedding light on potential intervention strategies and tailored educational approaches.

## 3. Clinical Challenges

### 3.1. Psychiatric Disorders

Earlier studies have highlighted worries about the heightened susceptibility of individuals with KS to psychiatric conditions like depression, anxiety, schizophrenia, and other psychotic disorders, when compared to the general population [40,41]. Nonetheless, it is worth noting that these studies have often been constrained by limited sample sizes. A study examining hospital admissions and discharge diagnoses among individuals with XXY in Denmark (*n* = 832) compared to an age-matched control group (*n* = 4033) discovered that those with XXY faced a higher relative risk of hospitalization for psychiatric disorders (hazard ratio: 3.65), especially for psychoses (hazard ratio: 4.97) [42]. Collectively, sex chromosome aneuploidies are recognized as contributing risk factors for the onset of psychosis [43,44,45]. This assertion is corroborated by observations of shared structural and functional brain irregularities between individuals with KS and schizophrenic patients, as well as comparable performance levels in cognitive assessments.

Psychiatric assessments and observations conducted on a group of 51 boys with KS revealed that nearly half of them (45%) exhibited psychotic symptoms, primarily auditory hallucinations and paranoid delusions [46]. Additionally, four participants (8%) met the criteria for a psychotic disorder not otherwise specified as per the Diagnostic and Statistical Manual of Mental Disorders (DSM 4), Text Revision. Two other boys received diagnoses of either schizophrenia or schizoaffective disorder. Furthermore, twelve boys (24%) in the study had encountered episodes of depression [46]. In light of these findings, it is imperative to recognize the multifaceted psychiatric challenges faced by individuals with KS, underscoring the importance of comprehensive and tailored support strategies to address their diverse mental health needs.

Alongside these investigations, several prospective longitudinal studies have examined the occurrence of psychiatric disorders in individuals with KS. A follow-up study of individuals (*n* = 19) diagnosed with KS at birth revealed a higher rate of psychiatric referrals (26%) compared to matched controls (9%) [47]. Similarly, another study by Bender et al. [48] found that adolescents with KS were more likely to receive psychiatric diagnoses (54%) compared to controls (14%). Among 13 adolescents with KS, seven received psychiatric diagnoses, with depression being the most common. Another follow-up study by Bender et al. [40] examined psychopathology in 11 young adults with KS using self-report measures and psychiatric interviews. They found that individuals with KS rated themselves as having fewer depressive symptoms and paranoid ideations than matched controls, but these differences were small and did not clearly indicate XXY-specific psychopathology. However, the level of psychopathology did help explain why individuals with KS reported higher social adaptation levels than those reported by psychiatrists. Individuals with more psychiatric symptoms tended to downplay symptoms expected to impact social adaptation. Even though the small sample size of this follow-up study limits its generalizability, it points to the possibility that individuals with XXY endure more psychiatric symptoms than they admit to.

Childhood manifestations of KS often entail behavioral challenges such as withdrawal and anxiety. Throughout their schooling and adolescence, individuals diagnosed with XXY commonly experience diminished self-esteem, heightened anxiety, mood disturbances, and difficulties in social integration [40]. The academic struggles, compounded by social exclusion, frequently precipitate anxiety and mood disorders in individuals with KS, albeit with considerable variability in symptom presentation [22,39]. An additional study identified a range of psychiatric conditions among a cohort of 51 boys with KS (mean age = 12.2 years old), including learning disorders (65%), a notable incidence of depressive episodes (24%), psychotic episodes (8%), and schizophrenia (2%) [49]. Furthermore, an increased risk for depression was observed in adolescents and adults with KS, with 68.8% of study participants reporting clinically significant levels of depressive symptoms, highlighting the need for routine screening and appropriate referral for evaluation and treatment [50]. The substantial number of participants reporting clinically significant depressive symptoms prompts us to consider whether these symptoms primarily stem from KS itself, secondary consequences of living with KS, or a combination of both factors. Research conducted by van Rijn and colleagues [51] revealed that males with KS encounter challenges in understanding social and emotional cues, coupled with heightened emotional responses to stimuli, yet they struggle to identify and express these emotions compared to the general population. These findings suggest potential risk factors for depressive symptoms or other psychiatric issues among individuals with KS, providing insights into the prevalence of depression observed in the study’s participants.

Overall, the research outcomes consistently highlight an increased susceptibility to psychiatric disorders in individuals with KS, often evaluated via psychiatric assessments. Despite certain studies facing constraints related to sample size, the prevalence of psychiatric conditions detected markedly surpasses that found among males in the broader population.

### 3.2. Social Impairments

KS has been implicated in social impairments that significantly impact daily functioning and life outcomes for affected individuals. Boys diagnosed with KS have been characterized as displaying shy and withdrawn behavior, frequently accompanied by notable deficiencies in social functioning. Research studies have documented social dysfunction in 42–47% of boys with KS [52,53]. Individuals affected by KS often encounter challenges in various aspects of social cognition, including perceiving, comprehending, and conveying social signals, particularly exhibiting deficits in interpreting facial expressions and affective nuances in tone of voice. These challenges can contribute to limited social support networks, difficulties in academic settings, struggles with employment acquisition and retention, and obstacles in forming intimate relationships. Understanding these social dysfunctions in younger populations is crucial for developing effective cognitive-behavioral interventions or training programs, a need that is currently unmet for individuals with KS.

Although the precise mechanisms underlying these social adaptation difficulties remain poorly understood, recent research suggests that individuals with KS may experience deficits in social cognition. Social cognition encompasses the ability to perceive, comprehend, and convey social cues. For instance, many boys with KS encounter challenges related to theory of mind (ToM), which involves interpreting the mental states, intentions, and emotions of others [54]. Additionally, KS has been linked to difficulties in interpreting social cues such as gaze direction, vocal tone, and facial expressions [54,55]. Evaluating the specific cognitive deficits in individuals with KS that contribute to social–emotional dysfunction is essential for developing targeted interventions to address these challenges. Van Rijn et al. [51] investigated social cognitive deficits in boys and men with KS finding that difficulties increased with the complexity of social information processing, particularly in interpreting facial expressions of emotion. The findings suggest that routine assessment of social cognitive functioning and targeted interventions may be beneficial for individuals with KS to address their specific social cognitive challenges.

### 3.3. Further Challenges

Studies have reported retinal dysfunction and vision impairment in individuals with KS [56,57]. Moreover, dental development may be influenced, resulting in taurodontism [58]. Additionally, KS patients may exhibit alterations in cardiac rhythm integrity, along with a high prevalence of cardiometabolic risk factors, such as elevated fasting triglycerides, low HDL cholesterol, and increased waist circumference in adolescents with KS compared to controls [59,60,61]. Nonetheless, these observations are predominantly based on small cohorts of KS patients and necessitate further validation.

The personal impact and challenges associated with living with this syndrome remain poorly understood. In a qualitative analysis, individuals aged 14 to 75 years old with KS discussed the hurdles they encounter in life [62]. More than half of the respondents, in response to open-ended questions, expressed difficulties in locating healthcare providers well-versed in XXY, often recounting prolonged diagnostic journeys and experiencing relief upon receiving a diagnosis. These individuals actively sought support to navigate their challenges and sought acknowledgment of the positive aspects of living with KS. Further exploration into the personal experiences of individuals with KS is warranted to gain a comprehensive understanding of their needs and experiences.

## 4. Discussion

**Figure 1:**
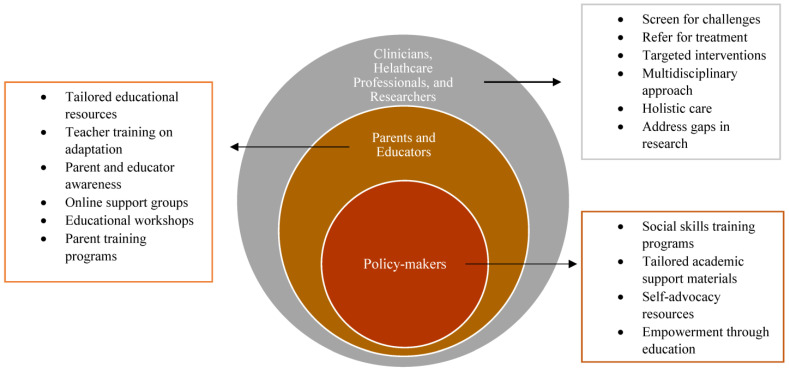
A multidisciplinary approach to KS management, emphasizing the collaboration among various stakeholders. It showcases the involvement of clinicians, healthcare professionals, researchers, educators, parents, and policymakers in addressing the diverse needs of individuals with KS across different domains, such as medical, psychological, educational, and social. This collaborative effort underscores the importance of holistic care and support for individuals with KS to optimize their well-being and quality of life.

This review offers a comprehensive overview of the neuropsychological profiles of children and adolescents with KS, synthesizing the key findings from existing research studies. Professionals across clinical, research, and educational domains can utilize this information to gain deeper insights into the cognitive and behavioral challenges experienced by individuals with KS. Understanding the neuropsychological deficits associated with KS is crucial for effective clinical practice. Therefore, healthcare providers, educators, parents, policymakers, and other stakeholders should recognize the potential impacts of executive dysfunction, language deficits, psychiatric disorders, social impairments, and other challenges on the overall well-being of individuals with KS (see Figure 1). This knowledge can inform the development of comprehensive assessment and intervention strategies tailored to the unique needs of each individual.

### 4.1. Clinicians, Healthcare Professionals, and Researchers

Given the susceptibility of the pediatric population with KS to psychiatric conditions [41,44,46,49,50], routine screening for neuropsychological challenges, including psychiatric disorders and social impairments, is recommended for individuals with KS. Clinicians should be prepared to appropriately refer patients for further evaluation and treatment as needed, ensuring that individuals receive timely and comprehensive care. Targeted interventions aimed at addressing neuropsychological deficits in KS can significantly improve outcomes for affected individuals. Cognitive-behavioral therapy, speech and language therapy, and androgen or psychopharmacologic treatments are among the strategies that may be beneficial [63,64]. The assessment and management of KS require a multidisciplinary approach involving a collaboration between clinicians, psychologists, educators, and other healthcare professionals [65]. By working together, professionals can provide holistic care that addresses the complex needs of individuals with KS across cognitive, emotional, and social domains. Despite the advances in understanding the neuropsychological aspects of KS, there are still gaps in the existing literature. Further research is needed to investigate the underlying mechanisms of neuropsychological deficits in KS and to evaluate the effectiveness of intervention strategies. Clinicians and researchers are encouraged to explore these research directions to enhance our understanding and improve outcomes for individuals with KS.

### 4.2. Educators and Parents

Educators and parents should work collaboratively to implement interventions and support individuals with KS in reaching their full potential. Educational resources for children and adolescents with KS can be developed and shared across educators and parents to address their specific needs across various domains, including cognitive, academic, social, and emotional. Resources for teachers on how to adapt classroom instructions to accommodate the diverse learning styles of students with KS should be developed. For example, implementing visual aids, providing extra time for assignments, or using assistive technologies can help support their learning. Educational brochures have proven effective in raising awareness and disseminating information, with teachers reporting their contribution to informing parents and shaping school practices in special education contexts [66,67]. Therefore, informational brochures containing basic information about KS, its symptoms, and common challenges faced by individuals with the condition can be distributed to parents and educators to increase awareness and understanding of KS. Additionally, providing case studies or real-life scenarios of successful inclusion practices in school settings can offer practical insights for educators and parents on how to support children with KS effectively. Further, online support groups or forums specifically for parents of children with KS and for individuals with KS themselves can provide a space for sharing experiences, asking questions, and offering support to one another. These platforms can also serve as avenues for sharing best practices and success stories related to inclusion and support for individuals with KS.

A recent study involving parents of KS recommended that communication regarding KS diagnosis should be initiated before the age of 18 years old, facilitated by a multidisciplinary team comprising endocrinologists, psychologists, geneticists, and parents [65]. Moreover, the information provided should encompass not only fertility and hormonal aspects, but also address metabolic and cognitive features [65]. Based on these findings, it is recommended that educational workshops or webinars for parents and educators should be organized to learn more about KS and how to support children and adolescents with the condition. Topics could include understanding the neuropsychological aspects of KS, effective teaching strategies, and promoting social skills development. Finally, parent training programs can be offered so that parents of children with KS can learn about effective parenting strategies, advocating for their child’s needs, navigating the educational system, and accessing support services. Providing concrete examples and practical strategies can enhance the effectiveness of these initiatives in promoting the inclusion and support of individuals with KS in educational settings.

### 4.3. Policymakers

Social impairments in individuals with KS have been widely acknowledged [51,52,53,54]. Considering the promising outcomes of social management training observed in adults with KS, it is advisable to introduce tailored social skills training programs for children and adolescents diagnosed with KS [68,69]. These programs can focus on teaching social communication skills, perspective-taking, emotional regulation, and building positive relationships with peers. Academic support materials tailored to the learning needs of individuals with KS should be developed. This could include visual aids, mnemonic devices, and other tools to aid memory and learning. Finally, self-advocacy resources and tools to help children and adolescents with KS become self-advocates for their own needs. This could include guidance on how to communicate with teachers and peers, seek accommodations, and access support services. Overall, educational resources for children and adolescents with KS should aim to empower individuals with the knowledge, skills, and support they need to thrive academically, socially, and emotionally. By addressing their specific needs and challenges, these resources can contribute to improved outcomes and quality of life for individuals with KS and their families.

## 5. Conclusions

Despite being recognized for over 80 years, KS still presents significant diagnostic hurdles, leading to frequent misdiagnoses or undiagnosed cases. In conclusion, this review provides a comprehensive examination of the neuropsychological profiles of children and adolescents diagnosed with KS, synthesizing the key findings from existing research studies. By synthesizing the available literature and delineating the limitations and implications of existing research findings, this review bridges the gap in understanding the cognitive and behavioral characteristics of children and adolescents with KS. The review underscores the multifaceted challenges faced by individuals with KS across various domains, including executive dysfunction, language impairments, psychiatric disorders, social impairments, and other associated difficulties.

Moving forward, further research is warranted to elucidate the underlying mechanisms of neuropsychological deficits in KS and to develop targeted interventions to address these challenges. Additionally, the continued exploration of the personal experiences of individuals with KS is essential to inform comprehensive care approaches that address both the medical and psychosocial needs of individuals with the condition and their families. Notably, the review highlights the need for a multidisciplinary approach involving clinicians, researchers, educators, parents, and policymakers to address the complex needs of individuals with KS effectively. Moreover, the review underscores the significance of educational resources, parental support programs, and social skills training initiatives tailored to the specific needs of individuals with KS. By increasing the awareness, understanding, and support for individuals with KS, these efforts can contribute to improved outcomes and quality of life for affected individuals and their families.

## Figures and Tables

**Table 1:** A summary of symptoms of KS.

Physical, Reproductive, and Endocrine Symptoms	Lannguage Deficits	Executive Dysfunction	Social Impairments
Gynecomastia Small testes Impaired spermatogenesis Azoospermia Testicular failure Androgen deficiency Elevated gonadotropin levels	Delayed babbling, lexical, and syntactic abilities Decreased vocabulary and comprehension Impairments in complex language processing, expressive vocabulary, verbal memory, and expressive grammar Challenges in understanding metaphors and implied meanings Reading/writing difficulties Persistent phonological errors in speech production	Deficits in information processing speed Selective and sustained attention difficulties Response inhibition challenges Cognitive flexibility issues Conceptualization difficulties Problems with initiating, planning, and goal setting Lower working memory capabilities	Shy and withdrawn behavior Challenges in perceiving, comprehending, and conveying social signals Deficits in interpreting facial expressions and affective nuances in tone of voice Difficulties in academic settings and employment acquisition/retention Obstacles in forming intimate relationships

## Data Availability

No new data were created.
